# Measuring women's perceived ability to overcome barriers to healthcare seeking in Burkina Faso

**DOI:** 10.1186/1471-2458-12-147

**Published:** 2012-02-27

**Authors:** Béatrice Nikiema, Slim Haddad, Louise Potvin

**Affiliations:** 1 Département de Médecine Sociale et Préventive, Université de Montréal, C.P. 6128 succ. Centre-Ville, Montréal, Québec H3C 3J7, Canada

## Abstract

**Background:**

In sub-Saharan Africa, women must overcome numerous barriers when they need modern healthcare. Respect of gender norms within the household and the community may still influence women's ability to obtain care. A lack of gender-sensitive instruments for measuring women's ability to overcome barriers compromises attempts to adequately quantify the burden and risk of exclusion they face when seeking modern healthcare. The aim of this study was to create and validate a synthetic measure of women's access to healthcare from a publicly available and possibly internationally comparable population-based survey.

**Method:**

Seven questionnaire items from the Burkina Faso 2003 DHS were combined to create the index. Cronbach's alpha coefficient was used to test the reliability of the index. Exploratory factor analyses (EFA) and confirmatory factor analyses (CFA) were applied to evaluate the factorial structure and construct validity of the index while taking into account the hierarchical structure of the data.

**Results:**

The index has a Cronbach's alpha of 0.75, suggesting adequate reliability. In EFA, three correlated factors fitted the data best. In CFA, the construct of perceived ability to overcome barriers to healthcare seeking emerged as a second-order latent variable with three domains: socioeconomic barriers, geographical barriers and psychosocial barriers. Model fit indices support the index's global validity for women of reproductive age in Burkina Faso. Evidence for construct validity comes from the finding that women's index scores increase with household living standard.

**Conclusion:**

The DHS items can be combined into a reliable and valid, gender-sensitive index quantifying reproductive-age women's perceived ability to overcome barriers to healthcare seeking in Burkina Faso. The index complies conceptually with the sector-cross-cutting capability approach and enables measuring directly the perceived *access *to healthcare. Therefore it can help to improve the design and evaluation of interventions that aim to facilitate healthcare seeking in this country. Further analyses may examine how far the index applies to similar contexts.

## Background

Women seeking modern healthcare in sub-Saharan Africa (SSA) are faced with numerous supply-side and demand-side barriers. They have to pay out of pocket, a significant challenge when they have already to cope with poor living conditions [1-7] and travel long distances to health facilities, especially for specialized services which are only available in urban centers [3,8-10]. In addition, poor road conditions and the underdevelopment of public transport, coupled with a lack of money to pay for them, often make transportation to health facilities difficult to organize [6,11-14]. While the cited studies have documented the independent effects of these barriers on women's health-seeking behavior, this study focuses on capturing the overall burden they impose.

Beyond the mere existence of barriers, it is the ability to overcome them that most likely influences health-seeking behavior. Other things being equal, health service utilization in SSA is higher among wealthy women [15-20], probably because they are more able to cover healthcare costs. However, an economically poor African woman may still manage to obtain some healthcare if she has an effective safety net [7,21-23]. At household and individual levels, women's ability to mobilize the resources needed to obtain healthcare depends not only on their personal and their household's wealth, but also on household functioning and living arrangements. Due to gender norms, procuring money for healthcare may depend on the willingness of the husband or household head to allocate household resources for this purpose, if such resources are available. Furthermore, in some African societies women need to obtain their husband's consent or the consent of other decision-makers before visiting a health center; in certain circumstances, they may not be allowed to travel alone or may feel uncomfortable doing so. Some women are unable or not allowed to use common transportation means (a bicycle or a motorcycle), even when these are available and the women know where to go to obtain appropriate care [24-26]. Healthcare seeking behavior can be expected to be sensitive to these gender-related factors.

There is a widespread awareness that access to healthcare is difficult for most African women and may be further hampered by gender-related constraints [27-33]. However, the burden and risk of exclusion women face remain unquantified due to the lack of an appropriate instrument for measuring their ability to overcome barriers to healthcare. In current research practice, inequalities in access to healthcare and risk of exclusion are often indirectly estimated by comparing patterns of *achieved utilization *between socio-economic groups of individuals [34-37]. Although these indirect evaluations have contributed to identifying groups who likely fail to receive needed care, utilization-based estimations of disadvantage are potentially problematic for at least two reasons. First, having utilized is not always synonymous of abilities to afford healthcare because users may have engaged themselves into catastrophic expenses^1 ^to acquire care [23,38-41]. Second, non-utilization is not necessarily due to inability to use because individuals may *prefer alternative sources *of care [23,42]. In SSA, this may occur, for instance, when the health problem is thought to be solvable only by traditional healing [43-46].

Rather, *direct *evaluation of *abilities *is needed to gain a better understanding of healthcare seeking behavior. In particular, looking at women's own evaluation of their ability to overcome healthcare barriers should help distinguish between capability-related and preference-related influences. The importance of this distinction is highlighted in the *capability framework*. This conceptual guide starts from the premise that a fair assessment of people's actions and quality of life should focus on analyzing what they are able to do or to be (their capabilities) rather than on what they actually accomplish (their achievements) [47]. Focusing on achievements may lead to distorted jugement because achievements are outcomes of available possibilities on one hand, and of individual preferences on the other hand. Two people with comparable achievents may still have unequal advantages if they have unequal achivement opportunities from which they can choose one over another [47-49].

A source of publicly available and internationally comparable data, the Demographic and Health Surveys (DHS), has recently introduced seven questionnaire items aiming directly at barriers that women of reproductive age may face when seeking healthcare. The questions address difficulties related to getting permission, going alone, finding transport, preferring a female health worker and covering the costs [50]. The data collected in SSA have so far mainly been used in the official DHS country reports. Some of the reports evaluate each of the seven items individually, others collapse them into a single dichotomous variable, yielding the proportion of women reporting difficulties with at least one of the seven aspects of access to care (see for example [51-54]). We found only a single study [55] that combined the information from the seven items to create an index of "mother's personal barriers" from principal components analysis, unfortunately without providing further details.

In this study, we use the DHS questions from the most recent available survey of Burkina Faso [52]. Burkina Faso is a poor landlocked country in West Africa, characterized by, among other things, weakly developed transportation facilities and a public healthcare system with low geographical coverage, applying user fees, and lack of collective health financing systems and mechanisms to prevent exclusion from healthcare [56,57]. Qualified health professionals are found mainly in a few urban centers, and there is a general lack of these professionals. Rural areas, in which more than 80% of the country's population lives, are underserved. As in many other SSA countries, most women (74%) are illiterate[58], underrepresented in the labor market [59] and often economically dependent on their husbands. Households members, including spouses, do not usually pool their income [60]. There is gender-specific allocation of activities and responsibilities and women are generally expected to respect their husbands' authority [61,62], including when they need healthcare.

We combine the new DHS questions to develop a continuous synthetic index of the women's capabilities to access healthcare. We evaluate whether this index is a reliable and valid measure of women's perceived ability to overcome common barriers to seeking modern healthcare in the context of SSA.

## Methods

### The data

The DHS routinely collects data on mortality, family planning, nutrition, fertility and other reproductive health issues, as well as on maternal and child healthcare, in over 70 developing countries [50]. Starting in 1999, the DHS included four sets of gender-specific questions, one of which contains questions on the barriers experienced by women in accessing healthcare for themselves from modern health services. These new questions were recommended to the DHS by a Gender Expert Advisory Board [50].

Burkina Faso first administered the questions in 2003, during its third Demographic and Health Survey (DHS), to a sample of 12,477 women of reproductive age (15-49 years). The sample was selected to be representative at the national, regional and urban/rural levels. A two-stage probabilistic sampling process was used in which first communities were drawn, then households. All women of reproductive age in selected households were surveyed. From these, we first excluded 284 visiting women (2.3%), then randomly selected from the remaining 12,193 women one per household, thus restricting the analyses to 7260 women from 400 communities. The data had been collected through standardized face-to-face interviews in the respondent's home, generally in the respondent's spoken language [52]. Participating women gave their informed consent before data collection. We requested and obtained the authorization to use the data from Macro International, which coordinates the DHS.

#### Items used for the index

We conceived the index of perceived ability to overcome barriers to healthcare seeking as a latent construct measured by 7 items. Women were asked about the difficulties they may have to surmount before accessing healthcare. The questionnaire items were worded as follows:

"Many different factors can prevent women from getting medical advice or treatment for themselves. When you are sick and want to get medical advice or treatment, is each of the following a big problem or not a big problem?

1- *knowing where to go to seek care;*

2- *getting permission to go;*

3- *getting money needed for treatment;*

4- *distance to health facility;*

5- *having to take transportation;*

6- *not wanting to go alone;*

7- *concern that there may not be a female health provider" *[63]

For the purpose of our index, not a big problem (coded 1) was contrasted with having a big problem (coded 0). A woman who reports that an item is "not a big problem" may mean that she perceives having a small problem or not having a problem at all with that item. For simplicity, we replace "not a big problem" by "small problem" with the understanding that it covers also the absence of a perceived problem. Having a small problem is considered as indicative of a perceived better ability to overcome a given obstacle. It is expected that a greater ability to overcome barriers will result in having a small problem on more items.

### Evaluating reliability and construct validity

We evaluated the reliability by testing for internal consistency of the set of items by Cronbach's alpha. Internal consistency indicates the extent to which the 7 items focus on the same content. The Cronbach's coefficient alpha varies from 0 to 1. Generally, values of 0.9 or greater are considered excellent, 0.8 good, 0.7 acceptable and 0.6 questionable. Internal consistency is deemed unacceptable below 0.5 [64,65].

Construct validity was evaluated by assessing the factorial structure and the nomological validity of the index. We carried out an exploratory factor analysis (EFA) on one half of the data (sample A), which we obtained by taking a random selection of the 7260 women. The purpose was to determine whether the set of items stand together on a unidimensional factor, or whether it is possible to identify a meaningful set of underlying common factors. The second half of the sample (sample B) was subjected to confirmatory factor analysis (CFA) to test the validity of the factor structure suggested by the EFA.

A valid construct should have the same factorial structure in different contexts, such as rural vs. urban. Women likely face different kinds and amounts of healthcare-seeking problems in these two areas. The availability and geographical accessibility of health resources, not to mention transportation systems, are usually better in urban than rural areas. Therefore, in addition to cross-validating the factor structure in sample B, we tested whether the identified factor structure is reproduced when fit to urban and rural subsets of women. We performed the EFA, the cross-validation with CFA, and the CFA of urban and rural areas to establish the factorial validity of the index.

Nomological validity is a form of construct validity and is evaluated by showing that the construct under investigation and other constructs, theoretically expected to be related to the focal construct, are positively correlated [66]. In our case, perceived ability to overcome barriers to healthcare seeking (the focal construct) is theoretically expected to be positively correlated with living standard after controlling for residential area, marital status, education and age. The household wealth index provided in the DHS database is used as indicator of living standard. We tested the factorial model in the whole sample, while regressing the latent construct on area of residence (rural vs. urban), household socioeconomic status (quintiles of the household wealth index), and level of woman's education (no education, primary, secondary or university) and marital status (never, formerly or currently married).

Both the EFA and CFA, were performed with Mplus software (version 5.2). We used mean and variance-adjusted weighted least-squares (WLSMV) estimation and oblique rotations. These technical specifications were found in simulation studies as producing more trustworthy results when variables are categorical [67,68]. Since the data were hierarchically structured, with individual women being nested in clusters of communities, we used the complex survey data technique which provides standard errors and chi-square statistics corrected for the non-independence of observations. The sampling weights contained in the DHS datasets were applied.

Several goodness-of-fit measures were used to evaluate whether the proposed model reproduces the empirical correlations of the manifest indicators. The chi-square model fit test indicates the extent to which the proposed model fits the data better than a baseline model where only the intercept and residual variances of the endogenous indicators (manifest variables) are estimated. In other words, it tests the null hypothesis of equivalence between the predicted and observed variance matrices. Lower chi-square values indicate better fit. By contrast, the null hypothesis is rejected (the model does not fit the data) if the p-value is < 0.05. We also used the root mean square error of approximation (RMSEA), the comparative fit index (CFI) and the Tucker-Lewis index (TLI). For RMSEA, ≤0.05 indicates good fit, ≤0.08 acceptable fit, and > 1.0 poor fit. A CFI and TLI of greater than 96% indicates a good fit. RMSEA and CFI are especially recommended for categorical data with sample sizes exceeding 250 units [69]. For the CFA, we also assessed whether the unstandardized loadings were statistically significant based on the ratio of the estimate to standard error. The absolute value of this ratio would have to be > 1.96 and > 2.56 for the related loading to be considered statistically significant respectively at the probability levels of 0.05 and 0.01 [70].

## Results

### Descriptive

Table 1 presents selected characteristics of the women for the whole sample and for each of the two subsamples, whereas Table 2 presents the proportion of women who reported having "a small problem" in overcoming each of the seven potential health-seeking hurdles. The two subsamples have similar characteristics and similar item response patterns. Mean participant age was 29.1 years (standard deviation: 9.5). A large majority of the women (86%) were married or formerly married, and most of them (81%) had never attended school. The head of household was female in 8% (n = 602) of cases and 5% (n = 373) of participating mothers were themselves head of household (not shown in Table 1). Overall, one in five women (21.5%, n = 1560) perceived themselves as having small problems with all of the 7 barriers when seeking care for themselves, whereas 8% (n = 645) reported having big problems with 6 to 7 barriers. Table 1 shows that obtaining money for treatment was the most frequent hurdle, with only 36% of women (n = 2630) stating this was a small problem for them. By contrast, obtaining permission (84%) or not having a female health worker (84%) seemed less problematic.

**Table 1:** Selected characteristics for women in the whole sample and for each of the two subsamples; data from the Burkina Faso 2003 DHS

	whole sample		sample A		sample B		
	N = 7260		N = 3611		N = 3649		
	n	(%)	n	(%)	n	(%)	P-value*
***Characteristics of the women***							
*Age group*							0.261
*15-19*	1371	(18.9)	701	(19.4)	670	(18.4)	
*20-24*	1383	(19.0)	683	(18.9)	700	(19.2)	
*25-29*	1299	(17.9)	636	(17.6)	663	(18.2)	
*30-34*	971	(13.4)	466	(12.9)	505	(13.8)	
*35-39*	918	(12.6)	487	(13.5)	431	(11.8)	
*40-44*	698	(9.6)	335	(9.3)	363	(9.9)	
*45-49*	620	(8.5)	303	(8.4)	317	(8.7)	
*Highest educational level*							0.265
*Never attended school*	5898	(81.2)	2901	(80.3)	2997	(82.1)	
*Primary*	821	(11.3)	431	(11.9)	390	(10.7)	
*Secondary or higher*	541	(7.5)	279	(7.7)	262	(7.2)	
*Marital status*							0.858
*Never married*	1008	(13.9)	509	(14.1)	499	(13.7)	
*Currently married*	5985	(82.4)	2968	(82.2)	3017	(82.7)	
*Formerly married*	267	(3.7)	134	(3.7)	133	(3.6)	
*Quintiles of household wealth index*							0.592
*Poorest*	1451	(20.0)	702	(19.4)	749	(20.5)	
*Poorer*	1389	(19.1)	692	(19.2)	697	(19.1)	
*Middle*	1712	(23.6)	848	(23.5)	864	(23.7)	
*Richer*	1107	(15.2)	547	(15.1)	560	(15.3)	
*Richest*	1601	(22.1)	822	(22.8)	779	(21.3)	
*Type of residential area*							0.642
*Urban*	1640	(22.6)	824	(22.8)	816	(22.4)	
*Rural*	5620	(77.4)	2787	(77.2)	2833	(77.6)	

**Table 2:** Proportion of women who perceive to have small problem with each potential healthcare seeking hurdle; data from the Burkina Faso 2003 DHS

	whole sample		sample A		sample B		
	N = 7260		N = 3611		N = 3649		
Not a big problem...	n	(%)	n	(%)	n	(%)	P-value*
*Knowing where to go to get care *	5921	(81.6)	2958	(81.9)	2963	(81.2)	0.432
*Getting permission to go *	6098	(84.0)	3051	(84.5)	3047	(83.5)	0.261
*Getting money for treatment *	2630	(36.2)	1340	(37.1)	1290	(35.4)	0.122
*Distance to health services *	3933	(54.2)	1981	(54.9)	1952	(53.5)	0.238
*Having to take transport *	4334	(59.7)	2156	(59.7)	2178	(59.7)	0.987
*Not wanting to go alone *	5447	(75.0)	2709	(75.0)	2738	(75.0)	0.994
*Concern about not having a female health worker *	6091	(83.9)	3021	(83.7)	3070	(84.1)	0.585

* P-value for chi-square test comparing sample A and sample B

### Reliability

The raw coefficient of Cronbach's alpha was estimated at 0.75, indicating that the index has adequate internal consistency. All items have strong relationships with the underlying construct because the value of the Cronbach's alpha decreases as each item is deleted. The largest drop is observed when the item "have to take transportation" (α = 0.69) is deleted, and the smallest drop is observed with the item "concern there may not be a female health provider" is deleted (α = 0.74).

### Factor structure and factorial validity

The results of the EFA conducted on the subsamples are presented in Table 3. A threecorrelated-factors model has the best fit indices and therefore appears to best fit the data in sample A. By contrast, solutions with less than three factors clearly fail to fit the data. Based on the values of the loadings (≥ 0.50), small problems related to getting money and getting permission are loading on the same factor (socioeconomic barriers); small problems related to having to take transport and small problems related to the distance to health services load on the second factor (geographic barriers); small problems related to knowing where to go for care, going alone, or having a female health worker load on the third factor (psychosocial barriers). Some indicators present large cross-loadings (that is, ≥|0.30|), suggesting a complex factor structure (see loadings printed in bold).

**Table 3:** Results of the exploratory factor analysis on data from the Burkina Faso 2003 DHS

	1-factor solution*		2-factor solution*			3-factor solution*			
	loading	(residual variance)	loading		(residual variance)	loading			(residual variance)
	1		1	2		1	2	3	
**Items**									
Distance to health services	0.896	(0.197)	-0.017	0.967	(0.083)	**0.987**	-0.025	-0.002	(0.044)
Having to take transportation	0.876	(0.233)	0.027	0.883	(0.194)	**0.859**	0.039	0.022	(0.214)
Getting permission to go	0.797	(0.364)	0.973	-0.011	(0.064)	-0.011	**0.778**	0.**453**	(0.059)
Getting money for treatment	0.626	(0.608)	0.531	0.21	(0.553)	0.346	**0.592**	-0.006	(0.396)
Having a female health worker	0.59	(0.652)	0.315	0.371	(0.637)	-0.013	-0.122	**0.840**	(0.332)
Going alone	0.672	(0.548)	0.379	0.403	(0.528)	0.063	0.006	**0.798**	(0.297)
Knowing where to go	0.784	(0.386)	0.688	0.203	(0.335)	0.090	**0.471**	**0.509**	(0.329)
**Interfactor correlations**									
2 with 1			0.543			0.339			
3 with 1						0.609			
3 with 2							0.202		
**Model fit** **indices**									
Chi-square model (df)	732.428 (14)		370.129 (8)				4.899 (3)		
p-value	< 0.001		< 0.001				0.179		
CFI	0.939		0.969				1.000		
TLI	0.909		0.920				0.999		
RMSEA	0.119		0.112				0.013		

df: degrees of freedom; CFI: comparative fit index (fit if > 0.96); TLI: Tucker-Lewis Index (fit if > 0.96); RMSEA: root mean square error of approximation (fit if ≤ 0.08)

*sampling weights were applied, and the results take into account the hierarchical structure of the data

**Figure 1:**
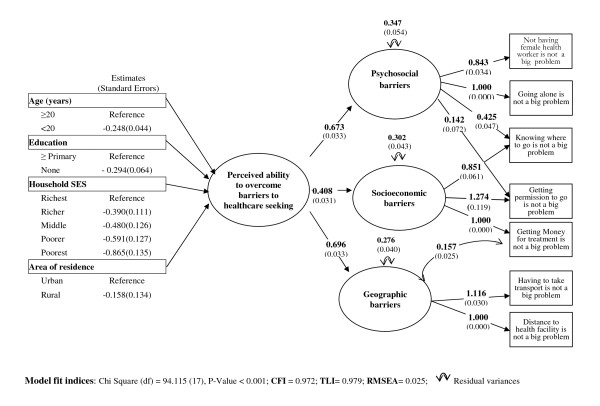
**Path diagram for the index of perceived ability to overcome barriers to healthcare seeking**. Shown are estimates and standard errors (in parentheses) from the final confirmatory model for the index of perceived ability to overcome healthcare seeking barriers in 7256 married women surveyed in the Burkina Faso 2003 DHS.

With the CFA, we first tested whether allowing the three factors identified in the EFA to load on a second-order factor fit the data in sample A. A complex factor structure was designed and the items "knowing where to go to seek care" and "getting permission to go" were allowed to load on both the first factor (socioeconomic barriers) and the third factor (psychosocial barriers). The second order 3-factor structure was reproduced with sample B, the whole sample, the rural sample and the urban sample. Table 4 shows fit indices for the different models tested in the CFA. All models perform well as shown by the fit indices; all of the unconstrained loadings are statistically significant (Figure 1).

**Table 4:** Fit indices for tested models in the confirmatory factor analysis on data from the Burkina Faso 2003 DHS

	sample A*†	sample B*†	urban*†	rural*†	whole*†
Sample size	3611	3649	1640	5620	7620
**Model chi-square**					
Value (df)	13.172 (7)	9.565 (7)	5.799 (6)	9.982 (7)	12.218 (7)
p-value	0.068	0.144	0.326	0.190	0.094
**CFI**	0.998	0.999	0.999	0.999	0.999
**TLI**	0.998	0.999	0.998	0.999	0.999
**RMSEA**	0.016	0.013	0.005	0.008	0.010

*sampling weights were applied and the results take into account the hierarchical structure of the data

†Model 1: second order, 3 factors, cross-loading allowed for "knowing where to go" and "getting permission"

df: degrees of freedom; CFI: Comparative Fit Index (fit if > 0.96); TLI: Tucker-Lewis Index (fit if > 0.96); RMSEA: root mean square error of approximation (fit if ≤ 0.08)

### Nomological validity

Figure 1 shows that the factor structure adequately fits the data in the whole sample. Estimated values for factor loadings and standard errors are placed on the arrows relating the latent variables to the indicators or relating the second-order factor to the first-order factors, with one factor loading constrained to unity. As indicated by the factor loadings, each indicator significantly contributes (at 5% alpha level) to the concept it represents. As expected for nomological validity, poor living standard is significantly negatively associated with the latent construct. Compared to women from households classed among the richest 20%, the *perceived ability to overcome barriers *gradually decreases from the richest group (estimate: -0.208; SE: 0.056), to the middle 20% group (estimate: -0.256; SE: 0.064), to the poorer (estimate: -0.315; SE: 0.063), and to the poorest 20% (estimate: -0.450; SE: 0.067). These results are controlled for participant age, marital status and education, as well as for location of residence (urban vs rural). The model has good fit indices, a finding that further supports that the model is nomologically valid.

## Discussion

### The index of the perceived ability to overcome healthcare seeking

Using data from the Burkina Faso 2003 DHS, this study evaluated the reliability and validity of a gender-sensitive index of the *perceived ability to overcome common barriers *to healthcare seeking among women of reproductive health. Our indicators of internal consistency showed that the index is reliable. Results also indicated that a single factor structure does not fit the data. Rather, the construct is best represented by 3 first-order latent factors (socioeconomic barriers, geographical barriers and psychosocial barriers) and a higher order latent factor (perceived ability to overcome barriers to healthcare seeking). The results support the validity of the index in the case of Burkina Faso because we were able to replicate the factor structure across different subsamples (sample A, sample B, rural area, urban area, whole sample), because the latent construct is as expected associated with household living standard, and because all tested models exhibit good fit indices.

The classification of items within the suggested factor structure is informative. Difficulties in getting money for healthcare is known to prevent many African households from getting needed care. Cash is not always available and users must often borrow or sell exchangeable goods [23,71-73]. In the current study, the fact that getting money, getting permission and knowing where to go load on the same factor can be viewed as an expression of the interrelationship between economic constraints and gender dynamics within the household and the community. In Burkina Faso, husbands have the duty to cover their wives' health expenses, and a previous study showed that permission to go for care may be refused, or even not requested, when household resources are scarce and the woman herself is lacking personal money to contribute [25]. The same study also showed that both getting money for treatment and getting permission may be problematic with poor inter-spousal or intra-household relations, and when the women's bargaining skills are poor. The indicators for this dimension therefore underscore the importance of gender-driven resource conversion factors postulated by the capability framework [47-49] in shaping perceived ability to overcome barriers to healthcare seeking.

The grouping of the items on the two other first-order latent factors is also logical. Not wanting to go alone and a concern about not having a female health worker may be linked to self-confidence and other psychosocial or cultural factors that render health service utilization uncomfortable to women. In this vein, it is understandable that knowing where to go to seek care loads with these items on the same factor (psychosocial barriers). Having to take transportation and distance to health facility obviously represent geographic factors. The fact that only two items load on this factor is a limitation because a minimum of three indicators per factor is recommended for model stability. However, a previous simulation study has shown that having a large sample size may compensate for a small number of indicators per factor [74]. In addition, all of the model fit statistics calculated suggest adequate model fit.

Moreover, poor living conditions were found to be associated with a lower perceived ability to overcome barriers: the lower the living standard, the lower the woman's position on the index of perceived ability to overcome barriers to healthcare. This result is an indication that the index has good construct validity since it has been repeatedly shown that low household socioeconomic status has negative effects on health service utilization in SSA, including in Burkina Faso [75-78].

The items reflect the fact of being (or not) in control of one's life, and the respondent is also able to weigh the importance of the control (having a big vs small problem). Both of these features help ensure that both gender and non-gender-driven experiences are captured. For instance, "obtaining permission to go" may apply to an employer, an insurance institution, the husband or some other household member. The need to ask for permission from an employer or insurance company is an administrative requirement imposed on all employees regardless of gender, yet the ease or difficulty in getting permission is subject to gender related-influences. The need to ask for permission from a husband or other household member is the most commonly encountered situation in SSA and is directly connected to social norms around marital relations. Given the weight of kinship and social ties in most parts of SSA [7,79,80], taking into account gender-related constraints improves the estimation of women's perceived ability to overcome barriers. That each item in the survey contributes significantly to capturing these constraints is supported by the high and statistically significant factor loadings for all items.

### Value added of the index

To the best of our knowledge, this is the first attempt to quantitatively capture in a single synthetic measure the relative weights of the various difficulties that women in sub-Saharan Africa face when seeking healthcare for themselves. Macro International, which coordinates the DHS MEASURE project, presents the seven questions as indicators of women's access to healthcare. As stated earlier, country reports usually present distributions for each item and for a synthetic dichotomous variable indicating the presence of any of the specified potential problems [81]. A compilation from the DHS MEASURE website shows that for countries that used the same 7 items to survey women within the past five years, eight out of 10 respondents in Cameroon (80%), the Congo Democratic Republic (86%), Guinea (82%), Malawi (79%), Niger (78%) and Rwanda (81%) reported having a big problem with at least one of the listed barriers [82]. A comparable rate (79%) was reported in the Burkina Faso 2003 DHS [52]. The proportions were 72% in Senegal, 66% in Mali, 60% in Tanzania, 60% in Madagascar and 55% in Lesotho [82]. While these reports indicate that accessing modern healthcare is somehow problematic for many African women, the index we created offers the opportunity to estimate the magnitude of the difficulty women perceive when needing healthcare.

Providing direct quantitative estimates for the magnitude of access capabilities can help public health policy-makers and managers to make more informed decisions. For instance, difficulties in access to healthcare have been made responsible for the persistent low-utilization of skilled care and the resulting high rates of maternal and child mortality and morbidity [32,83]. In response, several African countries are experimenting exemption policies in an attempt to stimulate utilization of maternal and child health services [84-87]. Having accurate insight into women's utilization capabilities at baseline and in subsequent evaluation processes should optimize the implementation, monitoring and impact evaluation of such policies. Effective measures to alleviate barriers should increase health service utilization by women with lower perceived abilities to overcome healthcare seeking barriers than by women with higher perceived abilities.

In her study, Gage [55] used principal component analysis (PCA) to generate an "index of maternal personal barriers," which was then used to predict the utilization of obstetric health services in Mali. Although PCA resembles factor analysis, it has a different aim. PCA reduces the number of relevant variables by decomposing their total variance into mutually *uncorrelated *components (unfortunately, Gage did not present her PCA results). By contrast, factor analysis more realistically decomposes their variance into (i) common *underlying *factors that may be *correlated *and (ii)unique variances of the indicator variables reflecting other aspects unrelated to the concept of interest [88]. The results of factor analysis are thus conceptually more meaningful.

Previous quantitative studies that could shed light on difficulties and abilities in accessing healthcare in SSA have mostly analyzed the economic burden of being sick and consuming healthcare, and their impacts more generally [7,71,89-92] or specifically on the ability to pay for care [23,93] or coping strategies [42,72,94]. However, these studies provide only limited information on women's experience because the unit of analysis is the household, not the individual within the household. While some studies tried to capture how gender relations within the household impact on healthcare-seeking behavior, most studies focused on women's behavior in resolving the healthcare needs of their children [7,26,95,96] rather than their personal healthcare needs [97]. Our study targets specifically women's personal healthcare needs and responds therefore to previous concerns over the limitations of the traditional focus on women as instruments for their children's health and wellbeing [98].

### Limitations and next steps

It is unfortunate that there were only 7 items to work with. The Advisory Group may have been subject to constraints on the number of questions to be included and who the survey could target (women of reproductive age) [50]. Some important aspects that could potentially generate additional burdens for healthcare-seeking women may thus have been left out; for example, constraints related to provider-client interactions and women's time budgets. Nevertheless, it is an advantage for future studies that these items are publicly available and possibly comparable between the DHS of similar of similar countries.

Another limitation is that the index represents abilities/barriers as *perceived *and may therefore be sensitive to cultural factors. Further studies are needed to evaluate how far our study results apply to similar countries, older women or even men. It would also be instructive to evaluate the index in different ethnic groups, as gender norms vary with ethnicity. When using DHS of other countries, one should benefit from additional items not included in the Burkina Faso DHS, such as time constraints and competing needs.

Contrary to countries such as Benin [99], Namibia [100], Nigeria [101] or Zimbabwe [102], which offered three ordinal response categories including "not a problem at all", Burkina Faso limited the response choices to only two categories ("big problem" and "not a big problem"). While the 3-level scale could enhance precision and discriminative power, the dichotomy has the advantage of simplicity. Notably, requiring women to focus on having big problem may mitigate bias due to response editing. Response editing occurs when respondents are concerned about social acceptability, when there is a social distance between the respondent and the interviewer, or when the respondent fears for the privacy of her input [[103,104], p 257-258]. For instance, a woman who is interviewed in presence of other members of the household may choose to underreport difficulties in getting money or getting permission because she does not want to embarrass her husband or the head of household.

Finally, the seven items target curative needs (being sick and wanting advice or treatment). It has already been reported that husbands are more willing to mobilize household resources for women's healthcare in case of pregnancy- and delivery-related complications or when a health problem is obviously severe or incapacitating [24-26,95,105]. It remains therefore open how far the index can capture difficulties in accessing healthcare for non-curative needs, such as contraception or ante-natal care.

## Conclusion

The questionnaire items on women's access to healthcare from the publicly available and internationally comparable DHS can be combined to create a gender-sensitive index that quantifies the perceived ability to overcome barriers to healthcare seeking. The index is reliable and valid for women of reproductive age in Burkina Faso. It can be useful for the design, implementation and evaluation of interventions to improve access to healthcare in this country.

The index complies conceptually with the sector-cross-cutting *capability approach *to evaluating people's freedom to achieve what they aspire. Hereby, the index enables us to directly measure perceived *access *to healthcare -- rather than to infer on access from achieved utilization, which fails to capture different degrees of difficulty in access. The index adds thus a qualitatively new tool that may help to enhance the relevance of interventions to improve access to healthcare.

Further studies (from other DHS or specific new surveys) may examine how far the factor structure applies in similar contexts. These studies could improve the validity and precision of the index by including additional items not available in the 2003 Burkina DHS.

## Endnotes

^1^According to Xu et al. [106] health expenditure is considered catastrophic "if a household's financial contributions to the health system exceed 40% of income remaining after subsistence needs have been met", p111.

## Competing interests

The authors declare that they have no competing interests.

## Authors' contributions

BN designed the study, carried out the analysis and prepared the first draft of the manuscript. LP and SH provided conceptual and methodological guidance. All of the authors contributed to the manuscript, and all of the authors read and approved the final version.

## Pre-publication history

The pre-publication history for this paper can be accessed here:

http://www.biomedcentral.com/1471-2458/12/147/prepub
